# Draft Genome Sequence of Clinical Strain TANI1 of *Streptococcus suis* Serotype 5 Isolated from a Bacteremia Patient in Japan

**DOI:** 10.1128/genomeA.00260-17

**Published:** 2017-05-04

**Authors:** Haruno Yoshida, Takayuki Wada, Daisuke Taniyama, Takashi Takahashi

**Affiliations:** a Laboratory of Infectious Diseases, Kitasato Institute for Life Sciences & Graduate School of Infection Control Sciences, Kitasato University, Tokyo, Japan; b Department of International Health, Institute of Tropical Medicine, Nagasaki University, Nagasaki, Japan; c Department of General Internal Medicine, Tokyo Saiseikai Central Hospital, Tokyo, Japan

## Abstract

*Streptococcus suis* is a swine pathogen that causes severe economic damage to the porcine industry. It occasionally evokes zoonotic infection in humans. Here, we report a draft genome sequence of a *S. suis* serotype 5 strain isolated from a bacteremia patient that was reported by Taniyama et al. (D. Taniyama, M. Sakurai, T. Sakai, T. Kikuchi, and T. Takahashi, IDCases 6:36–38, 2016, https://doi.org/10.1016/j.idcr.2016.09.011).

## GENOME ANNOUNCEMENT

*Streptococcus suis*, a swine pathogen, has been an agent of serious economic damage to the pork industry. This microorganism is a zoonotic pathogen that can be transmitted from pigs to humans, especially to those (with or without wounds) who handle raw pork or have close contact with infected pigs.

Thirty-three *S. suis* serotypes have been identified based on antigenic differences in their capsular polysaccharide (1). Serotypes 2 and 14 are prevalent among patients with *S. suis* infections, although other serotypes (1, 4, 5, 16, 21, and 24) are occasionally detected in humans. Three previous cases of patients infected with the serotype 5 have been documented: a patient in Thailand who developed spontaneous bacterial peritonitis following the consumption of raw pork (2), a pig farmer in Sweden who had an open wound and subsequently developed septic arthritis (3), and a pig farmer in the United States who developed arthroplasty infection and streptococcal toxic shock-like syndrome but had no open wounds (4). We reported a patient in Japan who had developed bacteremia from handling raw pork, and the *S. suis* serotype 5 isolate obtained from the patient contained a novel sequence type 752 (ST752), which was most similar to ST108 isolated from the heart and lung of diseased pig in Japan (5).

The draft genome sequence of *S. suis* serotype 5 clinical strain TANI1 (5) was obtained. The genomic DNA was purified from LB broth-cultured bacterium by lysozyme and proteinase K treatment, followed by phenol-chloroform extraction. A DNA sequencing library was prepared using the TruSeq Nano DNA library preparation kit (Illumina, Inc., San Diego, CA, USA), according to the manufacturer’s manual. The library was indexed and sequenced on Illumina MiSeq system using the MiSeq reagent kit version 3 (600 cycle) with other unrelated sequencing libraries. The draft genome sequence was automatically annotated using the Microbial Genome Annotation Pipeline (http://www.migap.org/) (6, 7).

The sequencing yielded 2,571,014 reads (762,758,497 bp), and *de novo* assembly of the genome was performed using CLC Genomics Workbench (version 7.5.2). Contigs shorter than 500 bp or with coverage lower than 100× were excluded. Finally, the assembled genome consisted of 54 contigs (2,207,658 bp, with a G+C content of 41.1%), with 265.3× average coverage. The genome contains 2,147 coding sequences, 43 tRNAs, and 3 rRNA loci, 1 phage-related region, and 5 incomplete phage elements. The genome harbors two antimicrobial resistance genes, *erm*(B) and *tet*(O), and some virulence-associated genes, including *mrp*, *sly*, *scpA*, *srtA*, and *dltA*. Another virulence-related gene, *epf*, was absent in the TANI1 genome. To date, the genome sequences of serotypes 1, 2, 3, 4, 7, 9, 14, 16, and 1/2 have been documented (8–11). The serotype 5 strain XS045 was reported to have been considered a live vaccine candidate (12). Genomic insights of TANI1 will support comparative genomics, pathogenicity analyses, and therapeutic and vaccine developments.

### Accession number(s).

The draft genome sequence has been registered in DDBJ database under the accession numbers BDMJ01000001 to BDMJ01000054.
